# Coding-Complete Genome Sequence of a Partitivirus Isolated from Pine Processionary Moth Eggs

**DOI:** 10.1128/MRA.00071-21

**Published:** 2021-02-25

**Authors:** Franck Dorkeld, Réjane Streiff, Carole Kerdelhué, Mylène Ogliastro

**Affiliations:** a CBGP, INRAE CIRAD IRD Institut Agro Université de Montpellier, Montpellier, France; b DGIMI, INRAE Université de Montpellier, Montpellier, France; KU Leuven

## Abstract

Two coding-complete nucleotide sequences of a partitivirus (family *Partitiviridae*) were discovered in transcriptomic data sets obtained from eggs of the Lepidoptera Thaumetopoea pityocampa. Each segment encodes a single open reading frame, and these two segments are predicted to encode an RNA-dependent RNA polymerase and a coat protein, respectively.

## ANNOUNCEMENT

Pine processionary moths (PPMs; Thaumetopoea pityocampa) are aggressive defoliators of pine trees in the Mediterranean basin (1). PPMs reproduce in summer and lay their eggs on needles at the tip of pine tree branches, where the caterpillars hatch and weave a large protective silken nest for the colony. Large transcriptome sequencing (RNA-Seq) data sets corresponding to several PPMs at all developmental stages (eggs to adults) were mined to determine the viral diversity in different populations from southern Europe. We describe here the full-length coding-complete genome sequence of a betapartitivirus (family *Partitiviridae*), associated with the eggs of a unique PPM population from Portugal that lays eggs in the spring (2).

**FIG 1:**
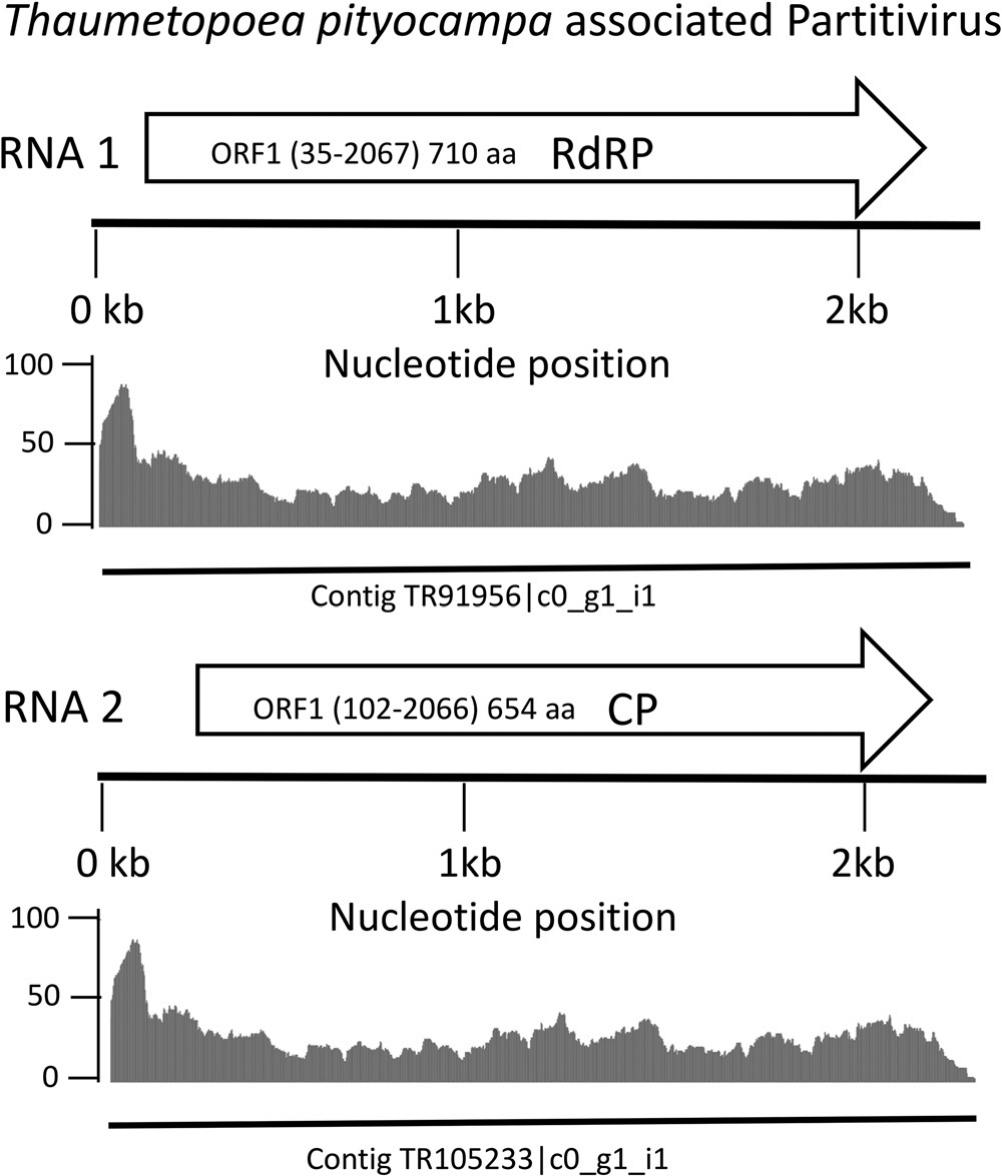
Genomic organization of the PPM-associated partitivirus. The complete major open reading frames (ORFs) predicted are represented by the arrows labeled RdRP (RNA-dependent RNA polymerase) and CP (capsid protein) and corresponding to RNA1 and RNA2, respectively. Matching reads mapped to the virus genomes are represented by a gray area with a scale that represents the coverage. The solid line at the bottom represents the contigs corresponding to the segments RNA1 and RNA2.

Viruses from the family *Partitiviridae* have a small and nonenveloped capsid protecting their bisegmented double-stranded RNA (dsRNA) genomes that are believed to be separately encapsidated (3). These viruses group into five genera infecting plants, fungi, and protozoa, with host-based clades and clusters within each genus (3), including viruses infecting mildew species, fungal causal agents of diseases, and major threats to agriculture (4). Eggs from *Thaumetopoea pityocampa* were collected in the region of Leiria, Portugal, in June 2012 (2, 4). The eggs were crushed, and total RNAs were extracted with TRIzol reagent. A library was constructed using the TruSeq stranded mRNA prep kit (Illumina) with an average insert size of 450 bp and sequenced in a lane of paired-end (PE) 2 × 100-bp reads on the HiSeq 2000 platform (5). We obtained 34,478,275 paired-end reads that were trimmed (Trimmomatic v.0.33) (6) and assembled into contigs (Trinity v.2.0.2) (7). Default parameters were used for all software unless otherwise specified. Viral contigs were identified by subjecting the contigs consecutively to BLASTX (E value, 1.e-10) and BLASTN searches against the viral RefSeq and nonredundant (nr) databases (NCBI), respectively. We identified two contigs of similar sizes (2,210 and 2,164 bp) made of the assembly of 754 and 830 reads, respectively, which represent 4.5% of the total viral reads in the sample. Both genetic elements are monocistronic; each is predicted to contain a single open reading frame (ORF). The first contig (2,210 bp; GC content, 39%) encodes a putative 710-amino-acid RNA-dependent RNA polymerase (RdRP); the second contig (2,164 bp; GC content, 40%) encodes a 654-amino-acid capsid protein (CP). Taxonomic sequence assignment showed that both contigs share the same closest relative, i.e., the Plasmopara viticola lesion-associated Partitivirus 7 (GenBank accession numbers MN556979.1 and MN556980.1), displaying 46% and 30% identity with the RdRP and CP, respectively. The contigs were named RNA1 and RNA2, according to the bisegmented genomic organization of this virus family (Fig. 1). According to the ICTV, taxonomic assignation in the family *Partitiviridae* is based on the RdRP homology, with a genus threshold of <24%. With 46% identity with the RdRP of the closest relative, two monocistronic contigs, and predicted proteins within the size range of this virus family, this PPM-associated partitivirus is likely a novel species in the genus *Betapartitivirus* (2). This virus was tentatively named *Thaumetopoea pityocampa*-associated partitivirus.

Members of the genus *Betapartitivirus* can infect plants and fungi, and the *Plasmopara viticola* lesion-associated Partitivirus 7 is an unclassified virus that infects the fungal causal agent of grape downy mildew (GenBank accession numbers MN556979.1 and MN556980.1 for RNA1 and RNA2, respectively). Although no fungal host can be associated with the novel partitivirus described here, mildew species are potential candidates, as they are commonly found in conifer forests, such as the powdery mildew that makes a white powdery substance on tree leaf surfaces. The most likely hypothesis is that infected fungi associated with pine trees have contaminated the surface of *T. pityocampa* eggs laid on the branches.

### Data availability.

The GenBank accession numbers are MT799182 and MT799183. The reads were deposited in the NCBI Sequence Read Archive (SRA) database under BioProject accession number PRJNA663237. The reads from the specific metagenome used here for virus reconstruction are registered with the SRA under accession number SRX9125299.
